# A High-Quality Draft Genome Assembly of the Black-Necked Crane (*Grus nigricollis*) Based on Nanopore Sequencing

**DOI:** 10.1093/gbe/evz251

**Published:** 2019-11-14

**Authors:** Chuang Zhou, Haoran Yu, Yang Geng, Wei Liu, Shuai Zheng, Nan Yang, Yang Meng, Liang Dou, Megan Price, Jianghong Ran, Bisong Yue, Yongjie Wu

**Affiliations:** 1 Key Laboratory of Bioresources and Ecoenvironment of the Ministry of Education, College of Life Sciences, Sichuan University, Chengdu, PR China; 2 Sichuan Key Laboratory of Conservation Biology on Endangered Wildlife, College of Life Sciences, Sichuan University, Chengdu, PR China; 3 College of Life Sciences, Huaibei Normal University, PR China; 4 Institute of Qinghai-Tibetan Plateau, Southwest Minzu University, Chengdu, PR China

**Keywords:** black-necked crane, nanopore sequencing, comparative genomics, positive selection, high-altitude adaptation

## Abstract

The black-necked crane (*Grus nigricollis*) which inhabits high-altitude areas has the largest body size of the world’s 15 crane species, and is classified as threatened by the IUCN. To support further studies on population genetics and genomics, we present a high-quality genome assembly based on both Illumina and nanopore sequencing. In total, 54.59 Gb Illumina short reads and 116.5 Gb nanopore long reads were generated. The 1.23 Gb assembled genome has a high contig N50 of 17.89 Mb, and has a longest contig of 87.83 Mb. The completeness (97.7%) of the draft genome was evaluated with single-copy orthologous genes using BUSCO. We identified 17,789 genes and found that 8.11% of the genome is composed of repetitive elements. In total, 84 of the 2,272 one-to-one orthologous genes were under positive selection in the black-necked crane lineage. SNP-based inference indicated two bottlenecks in the recent demographic trajectories of the black-necked crane. The genome information will contribute to future study of crane evolutionary history and provide new insights into the potential adaptation mechanisms of the black-necked crane to its high-altitude habitat.

## Introduction

**Fig. 1.**
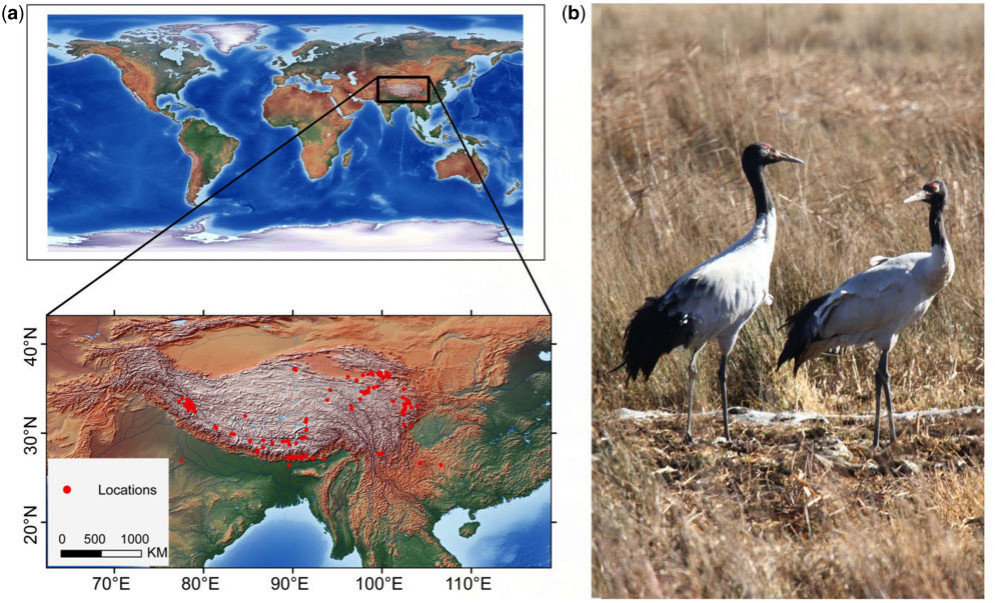
—Distribution and photo of the black-necked crane. (*a*) The latest distribution map of the black-necked crane in China based on specimen and field records in China. (*b*) The black-necked crane in the wild.

The black-necked crane (*Grus nigricollis*; Gruiformes: Gruidae) (fig. 1*a*) is threatened at the local, national, and global scales, and has subsequently been listed as a Class I protected animal in China, as vulnerable by the IUCN, and on Appendix I of CITES (Kong et al. 2011). The black-necked crane has the largest body size of the world’s 15 crane species and is the only crane that inhabits and reproduces at altitudes of ∼2,500–5,000 m asl (above sea level). The crane occurs mainly in China and India, with an additional nonbreeding population in Bhutan. The crane’s Chinese breeding range includes the Tibet, Qinghai, Xinjiang, Gansu, and Sichuan Provinces (fig. 1*b*; Qian et al. 2009; Song et al. 2014). During the winter, cranes in the Chinese range are mainly found in three regions: Northeastern Yunnan and northwestern Guizhou Provinces (eastern population); northwestern Yunnan (central population); and southern Tibet and Bhutan (western population) (Wu et al. 2009).

There have been an increasing number of avian genomes sequenced and compared, providing significant information including genomic signatures and genomic differences related to evolutionary adaptation (Zhang et al. 2014). To date, only genomes of gray crowned-crane (*Balearica regulorum*) (Zhang et al. 2014) and red-crowned crane (*Grus japonensis*) (GCA_002002985.1, deposited in NCBI) were sequenced in Gruidae. However, no high-quality Gruidae genome has been sequenced to date, and comparative genomics and analyses of genomic resources among Gruidae are thus severely limited. Furthermore, the vast majority of previously sequenced avian genomes was based on the Illumina sequencing technology, and many sequenced avian genomes had poor contiguity and contained thousands of scaffolds. Despite the relative completeness and correct assembly of the genes, fragmentation or underrepresentation is evident in TE (transposable element)-rich regions. Owing to the difficulty in repeat assemblies based on short-read technologies, the dynamics of TEs remain largely unknown. However, with the recent development of long-read sequencing technologies, such as Oxford Nanopore Technology (ONT) and Pacific Biosciences (PACBIO), we can obtain high-quality assemblies. It should be pointed out, however, that the error profile of the ONT platform contains random errors and a systematic issue when it comes to calling homopolymers (Johansson et al. 2018).

Recent technological advances have allowed previous studies to obtain high quality assemblies and have thus been able to explore the molecular genetic mechanisms underlying species’ environmental adaptations. Of particular interest for Gruiformes and black-necked crane ecology and conservation are the mechanisms underlying high-altitude adaptation (Qiu et al. 2012; Cai et al. 2013). The genetic mechanisms of high-altitude adaptation in the black-necked crane have never been investigated. It is possible that black-necked crane has adapted to high-altitude conditions through different genes or functional pathways.

There is considerable need for research in this area to advance general ecological knowledge of the species and to aid in conservation efforts as the crane’s high-altitude habitats become degraded and altered due to human impacts (e.g., climate change). So far, there is little genomic information about the black-necked crane, and the information in this study is crucial to the genetic management and conservation of black-necked crane populations. Moreover, it is highly important to further understand the molecular genetic mechanisms of black-necked crane’s high-altitude adaptation, and the aims of this study are to provide a high-quality genome assembly based on both Illumina and nanopore sequencing and to perform preliminary bioinformatic analyses.

## Materials and Methods

### Sample Collection and Sequencing

A muscle sample was collected from a wild female black-necked crane that died of natural causes in the Ruoergai National Nature Reserve in Sichuan Province, China. Genomic DNA was extracted using the DNA extraction kit following the manufacturer's protocol and prepared for DNA sequencing library construction. We used a whole genome shotgun approach on the Illumina HiSeq 2000 platform at Novogene (Beijing, China) to sequence the genome. We constructed the paired-end library with insert size of 230 base pairs (bp).

ONT libraries were prepared as follows: The genomic DNA sample in 49 µl was transferred to the Covaris g-TUBE, and the genomic DNA was sheared in a Covaris g-TUBE for 1 min at room temperature at the speed for the fragment size required. The g-TUBE was inverted and spun again for 1 min to collect the fragmented DNA. The 49 µl fragmented DNA was transferred to a clean 1.5 ml Eppendorf DNA LoBind tube. DNA CS (DCS) was thawed at room temperature, spun down, mixed by pipetting, and placed on ice. The NEBNext FFPE DNA Repair Mix and NEBNext End repair/dA-tailing Module reagents were prepared in accordance with manufacturer’s instructions, and placed on ice. In a 0.2 ml thin-walled PCR tube, mix (DNA CS: 1 µl, DNA: 47 µl, NEBNext FFPE DNA Repair Buffer: 3.5 µl, NEBNext FFPE DNA Repair Mix: 2 µl, Ultra II End-prep reaction buffer: 3.5 µl, and Ultra II End-prep enzyme mix: 3 µl) was mixed gently by flicking the tube, and spun down, then was incubated at 20 °C for 5 min and 65 °C for 5 min using a thermal cycler. AMPure XP beads were prepared for use and resuspended by vortexing. DNA sample was transferred to a clean 1.5 ml Eppendorf DNA LoBind tube. A total of 60 µl of resuspended AMPure XP beads were added to the end-prep reaction and mixed by pipetting, which then was incubated on a Hula mixer (rotator mixer) for 5 min at room temperature. A total of 500 µl of fresh 70% ethanol in nuclease-free water was prepared. The sample was spun down and pelleted on a magnet. The tube was kept on the magnet, and the supernatant was pipetted off. Beads were washed with 200 µl of freshly prepared 70% ethanol without disturbing the pellet. The 70% ethanol was removed using a pipette and discarded. The tube was spun down and placed back on the magnet. Any residual ethanol was pipetted off, and allowed to dry for ∼30 s. The tube was removed from the magnetic rack, and the pellet was resuspended in 61 µl nuclease-free water, which then was incubated for 2 min at room temperature. The beads were pelleted on a magnet until the eluate is clear and colorless. A total of 61 µl of eluate was removed and retained into a clean 1.5 ml Eppendorf DNA LoBind tube.

Adapter Mix (AMX) and T4 Ligase from the NEBNext Quick Ligation Module (E6056) were spun down and placed on ice. The Ligation Buffer (LNB) was thawed at room temperature, spun down and mixed by pipetting. Because of viscosity, vortexing this buffer is ineffective, so placement on ice immediately after thawing and mixing is needed. The Elution Buffer (EB) was thawed at room temperature, mixed by vortexing, spun down, and placed on ice. To enrich for DNA fragments of 3 kb or longer, one tube of L Fragment Buffer (LFB) was thawed at room temperature, mixed by vortexing, spun down and placed on ice. To retain DNA fragments shorter than 3 kb, one tube of S Fragment Buffer (SFB) was thawed at room temperature, mixed by vortexing, spun down and placed on ice. In a 1.5 ml Eppendorf DNA LoBind tube, the mix (60 µl DNA sample from the previous step, 25 µl LNB, 10 µl NEBNext Quick T4 DNA Ligase, and 5 µl AMX) was mixed gently by flicking the tube, and spun down. The reaction was incubated for 10 min at room temperature. The AMPure XP beads were prepared for use and resuspended by vortexing. A total of 40 µl of resuspended AMPure XP beads were added to the reaction and mixed by pipetting, which then was incubated on a Hula mixer (rotator mixer) for 5 min at room temperature. The sample and pellet were spun down on a magnet. The tube was kept on the magnet, and the supernatant was pipetted off. The beads were washed by adding either 250 µl LFB or 250 µl SFB. The beads were flicked to resuspend, then the tube was returned to the magnetic rack and the beads were allowed to pellet. The supernatant was removed using a pipette and discarded. The tube was spun down and placed back on the magnet. Any residual supernatant was pipetted off, and allowed to dry for ∼30 s. The tube was removed from the magnetic rack and pellet was resuspended in 25 µl EB, which then was incubated for 10 min at room temperature. The beads were pelleted on a magnet until the eluate is clear and colorless. A total of 25 µl of eluate was removed and retained into a clean 1.5 ml Eppendorf DNA LoBind tube. The prepared library is used for loading into the flow cell. Nanopore sequencing was conducted on the PromethION platform at Novogene (Beijing, China). We subsequently used the raw reads for the genome assembly.

### Genome Assembly and Evaluation

Before assembly, k-mer analysis was performed to estimate the genome size of the black-necked crane based on short insert size library reads. Long-read genome assembly was completed using the wtdbg2 software. As nanopore reads contain systematic errors in homopolymeric regions, we polished the consensus of the selected assembly three times with the nanopore reads as input to the Racon software (Vaser et al. 2017) and then two additional times using Illumina reads as input to the Pilon tool (Walker et al. 2014). Both tools were used with default parameters. BUSCO (Simão et al. 2015) analysis of conserved single-copy orthologues is widely used as a proxy for genome completeness and accuracy. We ran BUSCO on the black-necked crane genome assembly, along with 14 published avian genomes (supplementary table S1, Supplementary Material online).

### Repeat Annotation

We assessed and compared the repeat content of the black-necked crane genome and the other 14 avian genomes using RepeatMasker (http://www.repeatmasker.org/, accessed February 20, 2019). For further evaluation of the repetitive content of the black-necked crane genome and the other 14 avian genomes, we employed Krait (Du et al. 2018) to predict and characterize genome-wide microsatellite (SSR) loci, which can identify the loci that could be used for population genetic studies.

### Gene Prediction and Annotation

We identified the protein-coding genes (PCGs) in the black-necked crane based on the de novo and homology-based prediction. The de novo prediction was performed on the assembled genomes with repetitive sequences masked as “N” based on the HMM (hidden Markovmodel) algorithm. AUGUSTUS (Stanke et al. 2006) and GENSCAN (Burge and Karlin 1997) programs were employed to find coding genes using appropriate parameters. For the homology prediction, proteins of red jungle fowl (*Gallus gallus*), turkey (*Meleagris gallopavo*), zebra finch (*Taeniopygia guttata*), peregrine falcon (*Falco peregrinus*), and UniProt were mapped onto the black-necked crane genome using TBlastN (Altschul et al. 1997) with an E-value cutoff of 1E–5. To get the best matches of each alignment, the results which yielded from TBlastN were processed by SOLAR (Yu et al. 2006). Homologous sequences were successively aligned against the matching gene models via GeneWise (Birney et al. 2004). EVidenceModeler (EVM) (Haas et al. 2008) was used to integrate the above data and obtain a consensus gene set.

Functional annotation of the black-necked crane genes was undertaken based on the best match derived from the alignments to proteins annotated in the NCBI non-redundant (Nr), SwissProt, and TrEMBL databases. BLASTP tools were employed with the same E-value cut-off of 1E–5 for functional annotation. We retrieved the descriptions of gene products from Gene Ontology (GO) ID on the basis of the results of SwissProt. All genes were uploaded to KAAS (Moriya et al. 2007), a web server for functional annotation of genes against the manually corrected KEGG genes database by BLAST, in order to find the best match and associated pathway for each gene, using the bi-directional best hit (BBH) method.

### Phylogeny, Divergence, and Gene Family

We used orthoMCL (Li et al. 2003) to define orthologous genes from the nine avian genomes. Phylogenetic analysis was conducted based on one-to-one orthologous genes. Coding sequences of each one-to-one orthologous family were aligned by PRANK (Löytynoja and Goldman 2010), and were then concatenated to one sequence for each species for tree building. Modeltest was employed to select the best substitution model (Posada and Crandall 2005). We reconstruct the maximum likelihood (ML) phylogenetic tree with 1,000 bootstrap replicates using RAxML (Stamatakis 2014). Divergence time estimation was analyzed through the program Mcmctree within PAML (Yang 2007).

### Positive Selection Analysis

Estimation of the ratio of the rates of nonsynonymous to synonymous substitutions (*ω*) was performed based on the above alignments of one-to-one orthologous genes and the phylogenetic tree using the codeml program within PAML under the branch-site model (Yang 2007). We then performed a likelihood ratio test (LRT) and identified the positively selected genes (PSGs) by means of false discovery rate (FDR) adjustment with *Q*-values < 0.05.

### Demography Reconstruction

We used SAMtools to detect SNPs between diploid chromosomes (Li et al. 2009) and identified 835,930 heterozygous SNPs in the black-necked crane genome. On the basis of local SNP densities, we performed pairwise sequentially Markovian coalescent modeling (PSMC) (Li and Durbin 2011) analysis to model the demographic history of the black-necked crane. We inferred demographic history between 10 million and 10,000 years ago.

## Results and Discussion

### Genome Assembly

**Fig. 2.**
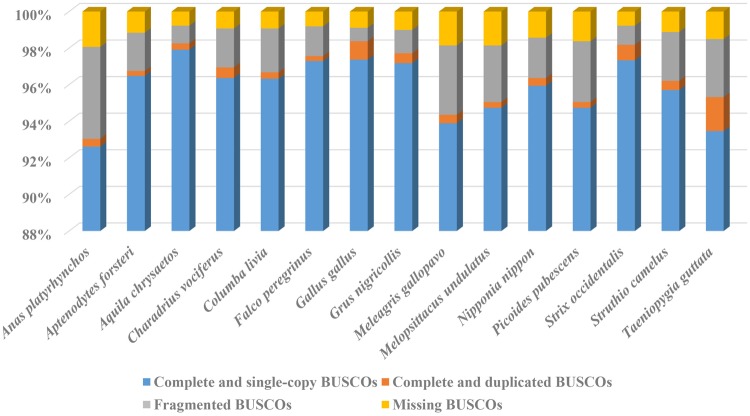
—Comparison of genome completeness of 15 birds.

After filtering out low quality and duplicated reads, we obtained 54.59 Gb (∼41.05-fold coverage) clean Illumina short reads (supplementary table S2, Supplementary Material online). We obtained ∼116.50 Gb (∼87.59-fold coverage) nanopore long reads that were not cleaned (supplementary table S2, Supplementary Material online). The genome size of the black-necked crane was estimated to be 1.33 Gb, which was larger than most reported avian genomes (Zhou et al. 2018, 2019). The total length of all contigs was 1.23 Gb, and the contig N50 length reached 17.89 Mb for the black-necked crane genome (table 1). BUSCO results showed that 97.7% of the eukaryotic single-copy genes were captured for the black-necked crane genome, and comparison with other 14 published avian genomes is shown in figure 2.

**Table 1:** Assembly Information of the Black-Necked Crane Genome

Parameters	Length of Contig (bp)	Number of Contig
Maximum length	87,834,618	—
N90	1,474,382	113
N80	4,016,951	64
N70	7,733,047	41
N60	14,171,613	30
N50	17,886,166	22
N40	20,086,248	16
N30	23,801,874	10
N20	28,462,007	5
N10	59,125,278	2
Total length	1,233,732,111	1,837
Number ≥ 2,000 bp	—	1,837

### Repeats in the Black-Necked Crane

**Fig. 3.**
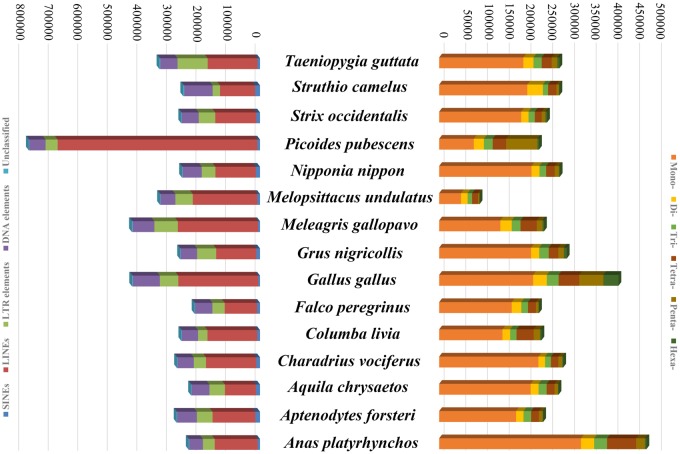
—Repeat annotation and SSR characterization of 15 avian genomes.

The GC content of the black-necked crane genome was ∼42.76%, similar to other bird species such as the Hainan partridge (*Arborophila ardens*), besra (*Accipiter virgatus*), and oriental scops owl (*Otus sunia*) (Zhou et al. 2018, 2019). We found that 100.01 Mb sequences (∼8.11% of the genome assembly) could be attributed to repeats in the black-necked crane genome that were not involved in the insertion of “N’s” into gaps. The percentage of long interspersed nuclear elements (LINEs), long terminal repeats (LTRs), short interspersed nuclear elements (SINEs), and DNA transposons were 4.78%, 2.68%, 0.13%, and 0.54% in black-necked crane genome. This estimation was higher than those reported for the saker (*Falco cherrug*) and peregrine falcon, scarlet macaw (*Ara macao*), turkey, and northern bobwhite (*Colinus virginianus*) genomes (Dalloul et al. 2010; Zhan et al. 2013; Seabury et al. 2013; Halley et al. 2014) but lower than the zebra finch (Warren et al. 2010) via RepeatMasker. It was reported that read-based scaffolding, which was involved in the insertion of “N’s” into gaps, led to the underestimation of genome-wide repetitive content (Seabury et al. 2013). Even so, there is a common feature of the black-necked crane, turkey, scarlet macaw, zebra finch, and northern bobwhite genomes, which is the high proportion of LINEs (Dalloul et al. 2010; Warren et al. 2010; Seabury et al. 2013; Halley et al. 2014) that are conserved across these divergent avian lineages. Comparison of repeats among 15 birds including the black-necked crane is revealed in figure 3.

Imperfect SSRs (1,181,394) were the most frequent type, followed by perfect SSRs (293,826), and finally compound SSRs (8,920) in the black-necked crane genome. In total, we identified 293,826 perfect SSR loci containing 1–6 bp sequence motifs. Comparison of perfect SSRs of 15 species is depicted in figure 3. The most frequent perfect SSRs were mononucleotide SSRs, with the highest frequencies of 171.56 loci/Mb and the highest densities of 2,645.78 bp/Mb, accounting for 72.04% of the total number of SSRs of the black-necked crane genome. The second most frequent SSRs were trinucleotide SSRs with a proportion of 7.48%. In contrast, dinucleotide, tetranucleotide, and pentanucleotide SSRs were less frequent, and the least was hexanucleotide SSRs, which only accounted for 2.29% of all of the SSRs. The most abundant motif categories were (A)n, (C)n, (AC)n, (AT)n, (AAAC)n, (AAT)n, (AG)n, (AAAT)n, (AAC)n, and (ACG)n, comprising 85.12% of all SSRs in the black-necked crane genome. Importantly, microsatellite genotyping can be utilized to assess population structure, gene flow, and covey composition within and between the black-necked crane populations. Thus, the resources described herein can be used for development of genetic markers for the black-necked crane.

### Gene Prediction and Annotation

We consequently found 17,789 genes predicted to be present in the black-necked crane genome. We found that 10,343 (58.14%) out of 17,789 identified PCGs were well supported by public protein databases (TrEMBL, SwissProt, Nr, GO, and KEGG) for the black-necked crane.

### Phylogeny, Divergence, and Gene Family

**Fig. 4.**
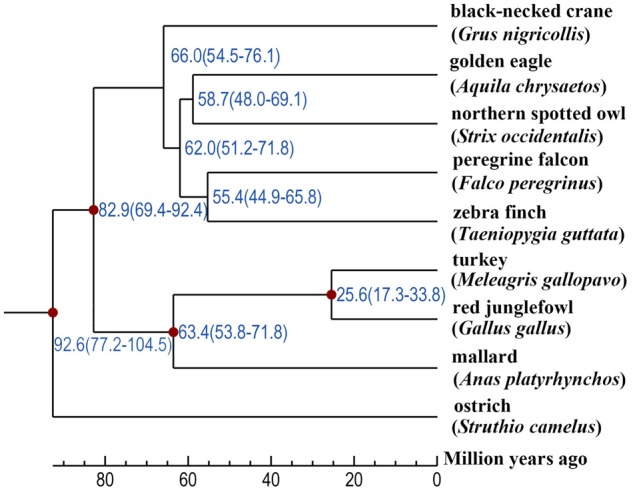
—Phylogenetic tree constructed using one-to-one orthologous genes.

We identified 14,585 gene families, of which 2,272 represented one-to-one orthologous gene families. In addition, there were 667 gene families specific to the black-necked crane. The ML phylogeny constructed based on the above one-to-one orthologous genes indicated that the black-necked crane was a sister group to the combination of the golden eagle (*Aquila chrysaetos*), northern spotted owl (*Strix occidentalis caurina*), peregrine falcon, and zebra finch, and it was most likely derived from a common ancestor ∼66.0 Mya (fig. 4). The series relationships within the nine birds were recovered as (((black-necked crane + (golden eagle + northern spotted owl) + (peregrine falcon + zebra finch)) + ((turkey + red jungle fowl) + mallard)) + ostrich), which was consistent with previous study (Jarvis et al. 2014).

### Positive Selection

**Fig. 5.**
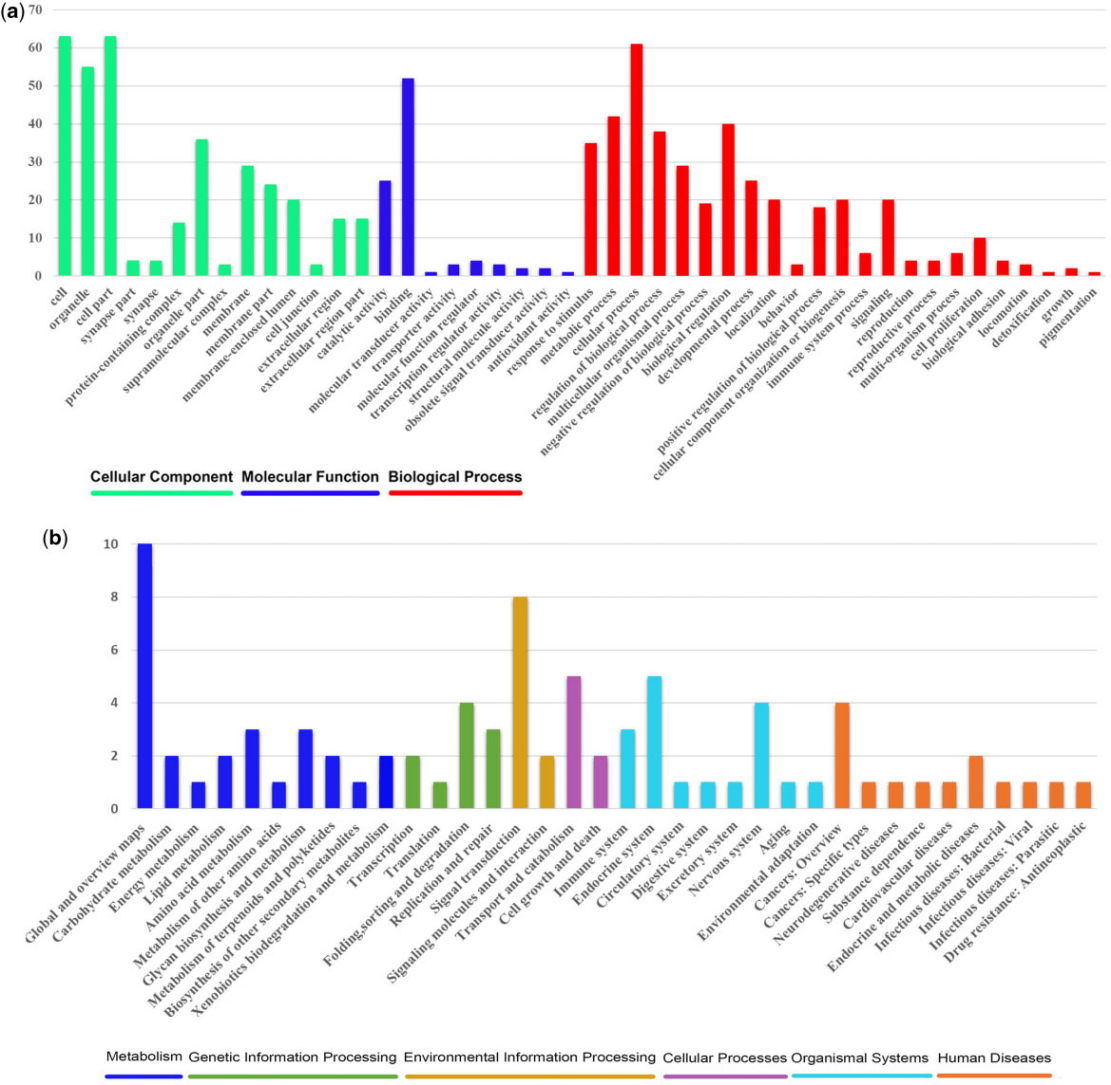
—Functional distribution of positively selected genes (PSGs). (*a*) Functional distribution of PSGs according to the KEGG pathway database. The *y*-axis illustrates the KEGG functional categories, whereas the number of genes in each category is plotted on the *x*-axis. (*b*) Functional distribution of PSGs according to the gene ontology (GO) database. The *y*-axis reveals the GO functional categories, whereas the number of genes in each category is plotted on the *x*-axis.

We found that 84 of the 2,272 one-to-one orthologous genes were under positive selection in the black-necked crane. The GO annotation classified the PSGs into three categories: Molecular functions, cellular components, and biological processes (fig. 5*a*). The distribution of GO annotations in different functional categories showed a substantial diversity of PSGs. We identified biochemical pathways represented by the PSGs. The KEGG annotation of the PSGs suggested that they were distributed in 36 pathways related to metabolism (13 genes), genetic information processing (nine genes), environmental information processing (nine genes), cellular processes (seven genes), organismal systems (seven genes), and human diseases (eight genes) (fig. 5*b*).

Within all PSGs, there were several genes relevant to high-altitude adaptation identified, such as *STC1*, *RNF8*, *RAD52*, and *PIAS4*. This evolution of genes may be important for the black-necked crane to survive in the high-altitude environment. This kind of adaptation has also been developed by other birds or mammals living at high altitude (Cai et al. 2013) A previous study found that *STC1* can induce adaptive responses to hypoxia in human cancer cells through the regulation of hypoxia inducible factor-1-alpha (*HIF-1α*) (Ma et al. 2015). It was reported that *RNF8* depletion sensitized cells to ionizing radiation, and *RNF8*-executed histone ubiquitylation played an important part in genome integrity protection (Mailand et al. 2007). The genes of the *RAD52* group played a crucial role in homology-dependent recombinational repair of double-strand DNA breaks that are induced upon exposure to ionizing radiation (Gangavarapu et al. 2007). Additionally, *PIAS4* was demonstrated to promote DSB (double-strand breaks) repair and confer ionizing radiation resistance (Galanty et al. 2009).

### Population History

**Fig. 6.**
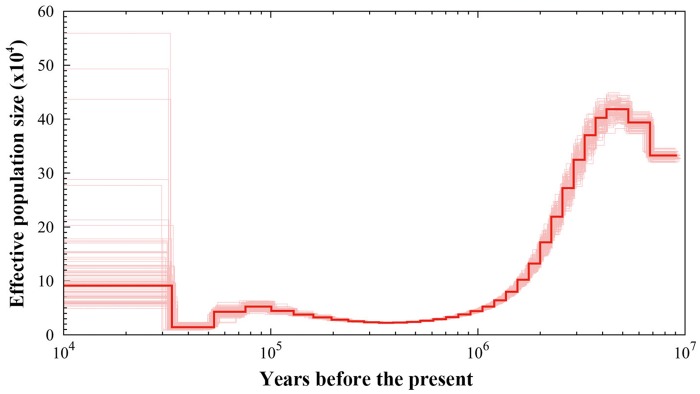
—PSMC inference of the black-necked crane population history based on the autosomal data. The central bold lines represent inferred population sizes. The 100 thin curves surrounding each line are the PSMC estimates that were generated using 100 sequences randomly resampled from the original sequence. The mutation rate on autosomes used in time scaling was estimated using red jungle fowl autosome data.

PSMC showed that the black-necked crane had experienced two bottlenecks (fig. 6). The effective population size decreased from ∼420,000 individuals ∼4 Mya to a minimum of 20,000 individuals 400,000 years ago. The black-necked crane populations then started to expand, eventually settling at around 50,000 individuals. However, the black-necked crane underwent a second bottleneck ∼75,000 years ago, and the population was again reduced to 12,000 individuals.

Here, we used a combination of Illumina and Oxford Nanopore reads to provide the high-quality complete genome assembly of the black-necked crane. Our study provides valuable genomic resources for studying the evolutionary adaptation and facilitating the long-term conservation and genetic diversity for this vulnerable species.

## Supplementary Material

Supplementary data are available at *Genome Biology and Evolution* online.

## Supplementary Material

evz251_Supplementary_Data

## Notes

**Data deposition:** This project has been deposited at the NCBI under the accessions SRR8705087, SRR8705088, SRR8708110, SRR8708111, SRR8708112, and SMUU00000000, and also at the Figshare under the link https://figshare.com/articles/genome_annotation_zip/7825403.
