# Supplementary material for: A High-Quality Draft Genome Assembly of the Black-Necked Crane (Grus nigricollis) Based on Nanopore Sequencing
Source: Genome Biol Evol. 2019 Nov 14;11(12):3332–40. doi: 10.1093/gbe/evz251 (PMC7145580; doi:10.1093/gbe/evz251)
Supplement: evz251_Supplementary_Data [file evz251_supplementary_data.docx]

**Table S1** Information of Avian Genomes Used in This Study

| Species | Family | Order | Assembly ID | Genome size (Gb) | Assembly quality (scaffold N50) (bp) |
| --- | --- | --- | --- | --- | --- |
| Mallard (*Anas platyrhynchos*) | Anatidae | Anseriformes | IASCAAS_PekingDuck_PBH1.5 | 1.11 | 1.23 |
| Emperor penguin (*Aptenodytes forsteri*) | Spheniscidae | Sphenisciformes | ASM69914v1 | 1.25 | 5.07 |
| Golden eagle (*Aquila chrysaetos*) | Accipitridae | Accipitriformes | Aquila_chrysaetos-1.0.2 | 1.19 | 9.23 |
| Killdeer (*Charadrius vociferus*) | Charadriidae | Charadriiformes | ASM70802v2 | 1.22 | 3.66 |
| Rock dove (*Columba livia*) | Columbidae | Columbiformes | Cliv_1.0 | 1.11 | 3.15 |
| Peregrine falcon (*Falco peregrinus*) | Falconidae | Falconiformes | F_peregrinus_v1.0 | 1.17 | 3.94 |
| Red junglefowl (*Gallus gallus*) | Phasianidae | Galliformes | GRCg6a | 1.23 | chromosome |
| Black-necked crane (*Grus nigricollis*) | Gruidae | Gruiformes | - | 1.23 | 17.89 |
| Turkey (*Meleagris gallopavo*) | Phasianidae | Galliformes | Turkey_5.0 | 1.13 | chromosome |
| Budgerigar (*Melopsittacus undulatus*) | Psittaculidae | Psittaciformes | Melopsittacus_undulatus_6.3 | 1.12 | 10.61 |
| Crested ibis (*Nipponia nippon*) | Threskiornithidae | Pelecaniformes | ASM70822v1 | 1.22 | 5.21 |
| Downy woodpecker (*Picoides pubescens*) | Picidae | Piciformes | ASM69900v1 | 1.17 | 2.09 |
| Northern spotted owl (*Strix occidentalis*) | Strigidae | Strigiformes | Soccid_v01 | 1.26 | 3.98 |
| Ostrich (*Struthio camelus*) | Struthionidae | Struthioniformes | ASM69896v1 | 1.23 | 3.59 |
| Zebra finch (*Taeniopygia guttata*) | Estrildidae | Passeriformes | Taeniopygia_guttata-3.2.4 | 1.23 | chromosome |

**Table S2** Sequencing Information of the Black-necked Crane Genome

| Libraries | Insert size | Total data (G) | Read length (bp) | Sequencing coverage (X) |
| --- | --- | --- | --- | --- |
| Nanopore | — | 116.5 | — | 87.59 |
| Illumina reads | 230 bp | 54.59 | 150 bp | 41.05 |
| Total | — | 171.09 | — | 128.64 |
